# Renin-Angiotensin System (RAS) Inhibitors in High-Risk Patients With Type 2 Diabetes and Cardio-Renal Risk

**DOI:** 10.7759/cureus.116699

**Published:** 2026-09-22

**Authors:** Mustafa J Ahmed, Aftab M Ahmad, Saf Naqvi, Khalid Ahmed, Aisha Ahmed, Zainab Naqvi

**Affiliations:** 1 Department of Nephrology, King's College Hospital London, Dubai, ARE; 2 Department of Endocrinology, Imperial College London Diabetes Centre, Abu Dhabi, ARE; 3 Faculty of Medicine, Pavol Jozef Šafárik University, Košice, SVK; 4 Faculty of Medicine, Andrzej Frycz Modrzewski Krakow University, Krakow, POL; 5 Department of Emergency Medicine, University College London Hospitals NHS Foundation Trust, London, GBR

**Keywords:** cardiovascular kidney metabolic syndrome, chronic kidney disease, microalbuminuria, rasi, type 2 diabetes

## Abstract

Background: Renin-angiotensin system inhibitors (RASi) are recommended as first-line therapy for patients with type 2 diabetes (T2D) and chronic kidney disease (CKD), particularly in the presence of hypertension or albuminuria. This study assessed the treatment patterns in a cohort of high-risk T2D patients.

Methods: This cross-sectional, retrospective study included 33,524 adults with T2D from the Imperial College London Diabetes Centre (United Arab Emirates). Demographic, clinical, and laboratory parameters (estimated glomerular filtration rate, urine albumin-to-creatinine ratio, and blood pressure (BP)), and medication usage were analysed. The primary outcome was the analysis of treatment patterns in patients undergoing RASi; secondary outcomes included prescribing trends in CKD risk categories and among hypertensive and albuminuric subgroups.

Results: Among 33,524 patients with T2D, mean age was 59.2 years, BMI 29.4 kg/m^2^, and 67.1% (n=22,508) of the patients were classified as KDIGO (Kidney Disease: Improving Global Outcomes) G1 and 73.3% (n=24,580) as A1 albuminuria. Overall, 35.3% (n=13,705) received RASi, while 64.7% (n=25,128) were treated with alternative therapies including SGLT2 inhibitors, GLP-1 receptor agonists, or finerenone. Among patients with albuminuria >3.5 mg/mmol, only 62% (n=4944) were prescribed RASi, despite meeting clear indications for treatment. In KDIGO-based risk stratification, only 39% (n=4406) of patients in the high and very high CKD progression risk categories were on RASi. Among the hypertensive subgroup (n=4,824; defined as BP >130/80 mmHg), 48.96% (n=2,362) were prescribed RASi, leaving over 50% of patients untreated with this guideline-recommended therapy. Furthermore, 7.46% (n=2,462) of the entire cohort had hypertension but were not receiving RASi. This gap was more prominent among those with coexisting albuminuria or CKD. These findings reflect under-treatment even among those at greatest risk of renal and cardiovascular events.

Conclusion: In patients with T2D and established renal or cardiovascular risk, treatment patterns remain variable in real-world practice. Less than half of hypertensive or albuminuric patients received RASi, highlighting a significant implementation gap. Addressing barriers to RASi prescription is essential to align real-world clinical practice with international guidelines and improve patient outcomes.

## Introduction

Chronic kidney disease (CKD) poses a high burden in patients with type 2 diabetes (T2D), with significantly higher complication rates, especially in patients with cardiovascular diseases (CVD) such as heart failure [1] and especially in patients with cardio-kidney metabolic disease [2]. Diabetic nephropathy (DN), a type of CKD caused by diabetes, is a leading cause of mortality among diabetic patients [3]. It is also called diabetic kidney disease (DKD), defined as either a reduced glomerular filtration rate or an elevated urine albumin-to-creatinine ratio UACR [4]. Globally, between 1990 and 2021, the burden of DN grew rapidly, with deaths increasing from 197.27 million to 571.29 million. If not controlled using effective public health measures, this trend might continue to accelerate, with projections estimating that by 2050 the global burden of DN could rise further, reaching nearly 954.8 million deaths [3].

The KDIGO Heat Map is a valuable tool utilised to evaluate the risk of kidney disease progression. It categorises individuals into different risk groups and assists in guiding treatment decisions [5]. The American Heart Association (AHA) defines the interrelated disease mechanisms linking diabetes, CKD, and CVD as cardiovascular-kidney-metabolic (CKM) syndrome [6]. The management of CKM syndrome necessitates addressing multiple risk factors, including elevated glycosylated haemoglobin (HbA1c), high blood pressure (BP), reduced estimated glomerular filtration rate (eGFR), elevated albumin-to-creatinine ratio (ACR), increased low-density lipoprotein cholesterol (LDL-C), and elevated body mass index (BMI) [6,7]. The increased risk of metabolic syndromes, such as CKD and coronary heart disease (CHD), is largely driven by unhealthy lifestyle changes [8], leading to mortality [9,10]. In a retrospective nine-year study, the cumulative incidence of CKD (stages 3 to 5) was reported to be 11.4%, and the primary risk factors included advanced age, a history of CHD, diabetes, and smoking [9].

For diabetic patients presenting with hypertension, major international guidelines advocate the use of renin-angiotensin system inhibitors (RASi), including ACE inhibitors or angiotensin receptor blockers (ARBs) [11, 12]. Both the American Diabetes Association (ADA) and KDIGO guidelines recommend the initiation of an RASi (ARB in T2D patients) with elevated uACR titrated to the maximum tolerated dose [13-15]. Further, RAS blockade decelerates the progression of diabetic kidney disease (DKD) and reduces cardiovascular risk [16]. However, a significant gap still remains between the guideline recommendations and real-world clinical usage, such as initiation and continuation of treatment [17].

There are four main pillars of treatment in patients with DKD, including sodium-glucose cotransporter-2 inhibitors (SGLT2i), non-steroidal mineralocorticoid receptor antagonists (MRAs), RAS blockade, and glucagon-like peptide-1 (GLP-1) agonists. Recent landmark studies show the benefit of these agents in patients who are already undergoing the maximum tolerated dose of RASi [18]. Among these options, RASi remains the cornerstone therapy for managing hypertension, CKD, and DKD [19]. The benefits of RASi have also been demonstrated in the RENAAL and IDNT trials, conducted in diabetic patients with hypertension and proteinuria [20,21]. However, a residual risk of disease progression remains, highlighting the additional benefits of add-on therapy with the above-mentioned agents. An additional treatment or the implementation of multiple therapies simultaneously may provide quicker improvements and better clinical outcomes [22]. In the DAPA-CKD study, 98% of patients treated with dapagliflozin were on RASi treatment [23]. In the EMPA-KIDNEY trial [24,25], 86% of patients treated with empagliflozin were also receiving concomitant therapy with a RASi. This combination is clinically meaningful, as a synergistic effect is often observed: RASi lower intraglomerular pressure, while SGLT2 inhibitors reduce hyperfiltration and improve metabolic control, together providing renoprotective benefits beyond glycaemic control [26]. Supporting this, an analysis reported a reduced benefit of SGLT2 inhibitors in 981 patients who were not on RAS blockade, highlighting the importance of combined therapy [27].

Similarly, patients with proteinuria and T2D underwent treatment with finerenone, on RASi (65% and 35% on ARBs and ACE inhibitors, respectively) in the FIDELIO and FIGARO-DKD trials [28-30]. Further, according to the pooled FIDELITY analysis of 13026 patients, the majority of the patients tolerated RASi, and the composite kidney outcome occurred in 15.3% of patients in the finerenone group while on RASi therapy [31]. Moreover, the FLOW study demonstrated a 24% reduction in primary renal outcomes in 97% of patients on concomitant treatment with GLP-1 receptor agonists and RASi [32]. Similarly, the CONFIDENCE study demonstrated that combination treatment with SGLT2 inhibitors and finerenone in patients receiving RASi (98.4%) reduced UACR by 40% [33]. Therefore, RASi remains the cornerstone and first-line treatment for patients with DKD in accordance with multiple international guidelines such as AACE [34], KDIGO [14], ADA [13], and ESH 12 [12]. Additionally, the KDIGO guidelines also recommend administering RASi at the maximum approved and tolerated dose [35]. Its varying usage likely plays a significant role in the treatment gap seen in managing the combined cardiovascular and renal burden in high-risk groups. Nonetheless, RASi have been associated with certain adverse effects, including hyperkalemia, acute renal failure, and notable reductions in blood pressure, which may contribute to variations in their use and non-adherence [36]. Furthermore, non-compliance with treatment may also be attributable to additional comorbid conditions [37], limited follow-up [38], and barriers related to insurance [39].

This study was undertaken to evaluate real-world prescribing patterns among high-risk individuals with T2D who present with established renal and CV risk factors and are undergoing RASi treatment. This study was designed to understand and identify the unmet clinical needs that can drive optimisation of clinical care and ensure closer alignment with both international and regional guideline recommendations. Ultimately, the purpose is to strengthen patient outcomes by promoting more consistent therapeutic strategies by understanding the prevalence and treatment patterns in T2D patients with established renal or CV risk.

## Materials and methods

Study design

This was a cross-sectional, retrospective analysis designed to descriptively evaluate real-world prescribing patterns in patients with T2D and established renal and CV risk, undergoing therapy with RASi. Data were collected from the Imperial College London Diabetes Centre (ICLDC) network across the United Arab Emirates. The final dataset included 33,524 eligible patients based on specific criteria. The inclusion criteria involved adults aged ≥18 years with a confirmed diagnosis of T2D. Further, patients with end-stage renal disease or a glomerular filtration rate of less than 15 ml/min/m2 were excluded.

Electronic health records were reviewed and anonymised prior to analysis. Extracted variables included demographics (age, gender, BMI), clinical measures (systolic and diastolic BP, recorded by trained auxiliary staff using standardised protocols), and laboratory parameters (HbA1c, LDL-C, eGFR, and uACR). Treatment data were collected for four key therapeutic classes that included RASi, SGLT2 inhibitors, GLP-1 RAs, and finerenone.

The primary outcome was to understand the prevalence and treatment pattern in T2D patients with established renal or CV risk undergoing treatment with RASi. The secondary outcomes comprised distribution of patients by CKD stage (G1-G5) and albuminuria category (A1-A3) according to KDIGO 2022 [14], proportion of RASi users across KDIGO risk strata, concomitant use of RASi with SGLT2 inhibitors, GLP-1 RAs, and finerenone, and association between RASi use and albuminuria (uACR >3.5 mg/mmol).

CKD staging was based on eGFR and albuminuria categories as per KDIGO 2022 guidelines [14]. A KDIGO heat map was generated to classify patients into low, moderate, high, and very high risk of CKD progression. Patients in high- and very high-risk categories were further analysed for CKM markers, including HbA1c, LDL-C, BMI, and BP.

Statistical analyses were performed using IBM SPSS Statistics for Windows, version 23 (IBM Corp., Armonk, NY, USA), Microsoft Excel (Microsoft Corporation, Redmond, WA, USA), and R version 4.3.0 (R Foundation for Statistical Computing, Vienna, Austria). Continuous variables were reported as means with standard deviations, while categorical variables were expressed as frequencies and percentages. Subgroup analyses were conducted for hypertensive and albuminuric patients. Patients with missing data for key variables (such as eGFR, uACR, BP) were excluded from the subgroup analyses. Baseline characteristics of patients with or without missing data were compared to assess for potential bias. No imputation was performed for missing values.

The study was approved by the Research Ethics Committee of the ICLDC, Abu Dhabi (Approval No. IREC102, dated August 7, 2024). All procedures adhered to the Declaration of Helsinki and the International Council for Harmonization-Good Clinical Practice (ICH-GCP) guidelines [40].

## Results

Patient characteristics

Out of a total of 33,524 patients with T2D included in the analysis, the mean age was 59.24 ± 12.49 years (range: 18-95 years). The mean BMI was 29.41 ± 4.41 kg/m². Mean systolic and diastolic blood pressures were 124.98 ± 15.57 mmHg and 72.85 ± 9.08 mmHg, respectively, with 14.39% (n = 4824) meeting criteria for hypertension (BP >130/80 mmHg).

Renal and metabolic parameters

The mean eGFR was 100.29 ± 26.61 mL/min/1.73 m², whereas the mean ACR level was 8.07 ± 30.16 mg/dL, and the mean LDL-C was 2.19 ± 0.97 mmol/L in this analysis. In this study cohort involving T2D patients, the mean HbA1c was 7.26 ± 1.34 %, and the mean microalbumin was 55.33 ± 219.24 µg/min. The comprehensive descriptive profile for the cohort according to each parameter is presented in Table 1.

**Table 1:** Demographics of the patient cohort ACR = albumin-to-creatinine ratio; BMI = Body Mass Index; BP = blood pressure; eGFR = estimated glomerular filtration rate; HbA1c = haemoglobin A1c; LDL = low-density lipoprotein; N = number of patients

N=33524	Minimum	Maximum	Mean	Std. Deviation
Age	18	95	59.24	12.49
Latest Haemoglobin	100.00	209.00	133.07	16.01311
Latest BMI	18.50	40.00	29.41	4.41034
Latest BP systolic	80.00	197.00	124.98	15.57620
Latest BP diastolic	50.00	117.00	72.86	9.08912
Latest eGFR	15.03	149.99	100.29	26.61863
Latest Microalbumin	.07	4400.00	55.33	219.24734
Latest Ferritin	1.10	8960.50	87.23	158.89033
Latest HbA1c	5.70	14.80	7.26	1.34690
Latest LDL	.10	8.24	2.19	.97782
Latest ACR	.05	499.27	8.07	30.16548

CKD staging and albuminuria categories

Based on KDIGO classification: 67.14% (n=22,508) of patients were in G1 (normal kidney function), 24.25% (n=8129) in G2, 4.77% (n=1599) in G3a, 2.19% (n=734) in G3b, and 0.78% (n=262) in G4. Albuminuria was categorised as A1 (<3.5 mg/mmol): 73.32% (n = 24,580), A2 (3.5-30 mg/mmol): 18.55% (n = 6219), and A3 (30-1000 mg/mmol): 5.23% (n = 1753).

A total of 7950 patients demonstrated albuminuria >3.5 mg/mmol, consistent with established nephropathy (Table 2).

**Table 2:** Percentage of T2D patients with ACR>3.5 treated with SGLT2i, GLP-1, RASi and finerenone ACR = albumin-to-creatinine ratio; T2D = type 2 diabetes; SGLT2i = sodium-glucose cotransporter 2 inhibitors; GLP-1 RAs: glucagon-like peptide-1 receptor agonists; RASi: renin–angiotensin system inhibitors

Treatment modalities	Yes	No
SGLT2i	4795 (60%)	3155 (40%)
GLP-1	2189 (28%)	5761 (72%)
RASi	4944 (62%)	3006 (38%)
Finerenone	478 (6%)	7472 (94%)

Treatment patterns

Overall, 35.29% (n=13,705) of patients were prescribed RASi, while 64.71% (n=25,128) received other modalities, including SGLT2 inhibitors, GLP-1 receptor agonists, or finerenone (Table 3).

**Table 3:** Percentage of T2D patients treated with SGLT2i, GLP-1, RASi and finerenone GLP-1 RAs = glucagon-like peptide-1 receptor agonists; RASi = renin-angiotensin system inhibitors; SGLT2i = sodium-glucose cotransporter 2 inhibitors; T2D: type 2 diabetes

Treatment modalities	Frequency	Percentage (%)
SGLT2i	16706	49.83
GLP-1	7856	23.43
RASi	13705	35.29
Finerenone	566	1.69

Among patients with albuminuria >3.5 mg/mmol (n = 7950), 62% (n = 4944) were treated with RASi. Approximately 25% of the overall cohort had albuminuria, of whom 60% received RASi therapy (Figure 1). Among the hypertensive subgroup (n = 4824), 48.96% (n = 2362) were receiving RASi therapy.

**Figure 1:**
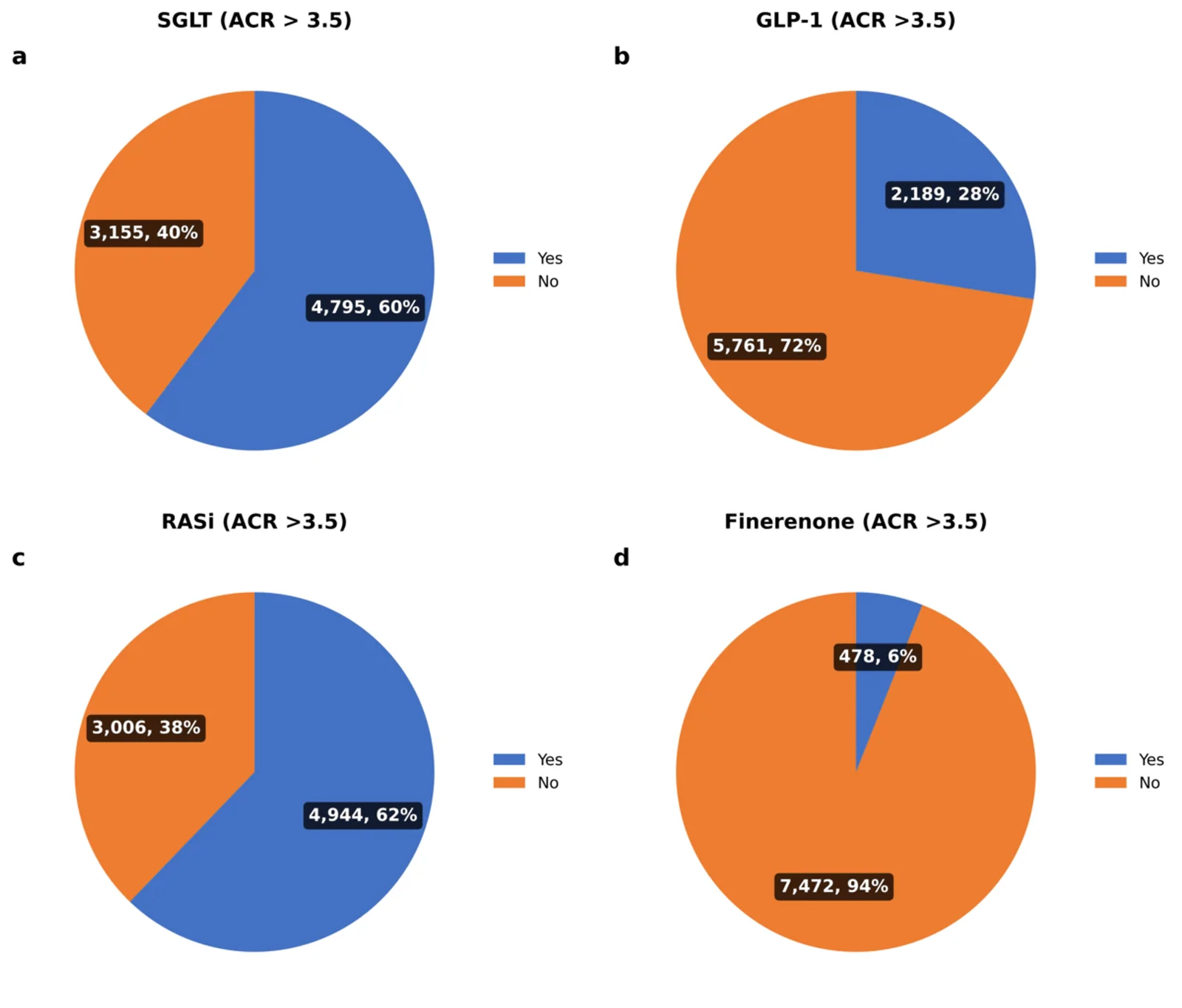
Patients with evaluated uACR undergoing treatment (a) SGLT-2 = sodium-glucose cotransporter 2; (b) GLP-1 = glucagon-like peptide-1; (c) RASi: renin-angiotensin system inhibitor; (d) finerenone uACR: urine albumin-to-creatinine ratio

However, 7.46% (n=2462) of the overall cohort had hypertension (BP > 130/80) and were not on RASi (Figure 2).

**Figure 2:**
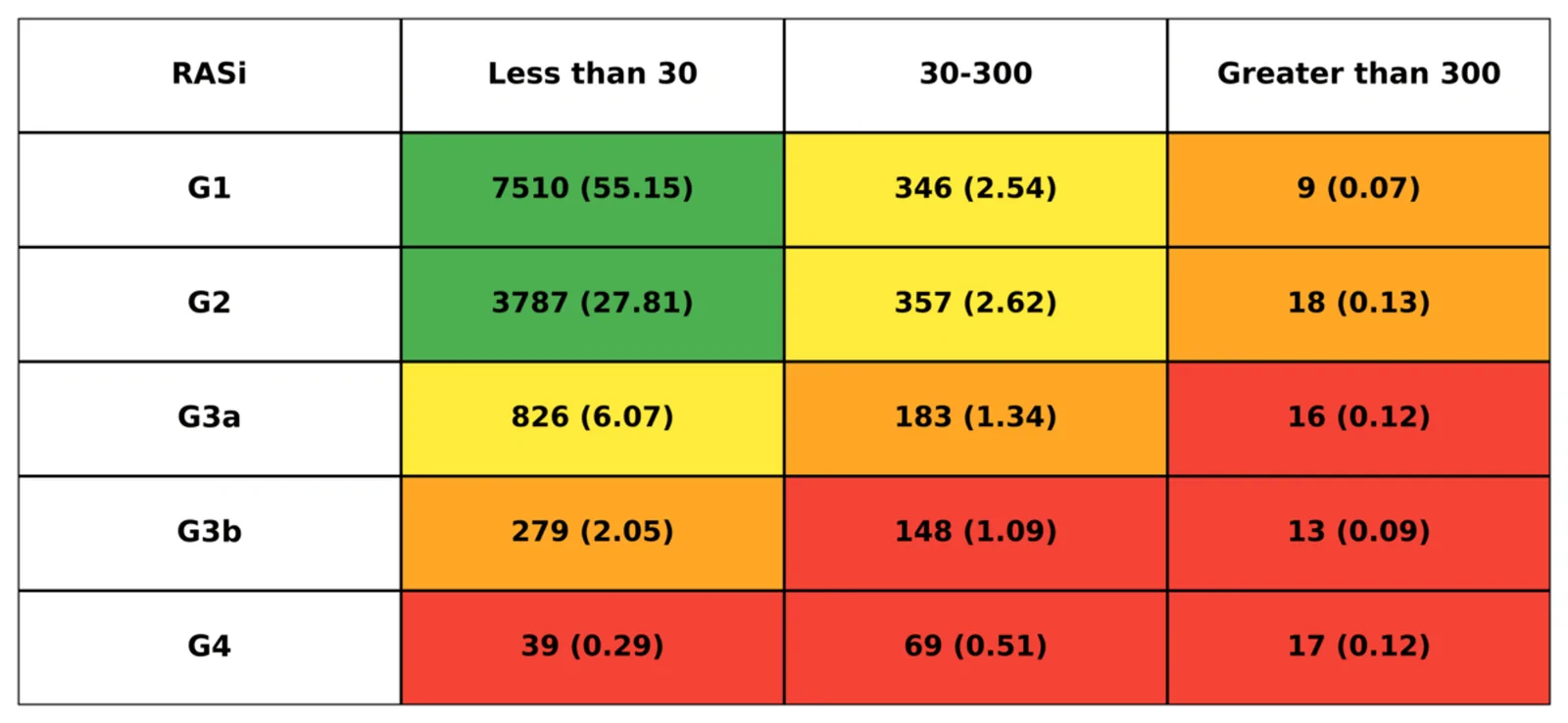
Risk stratification of diabetic patients undergoing RASi as per KDIGO heat map (n=13617) RASi = renin-angiotensin system inhibitors; KDIGO = Kidney Disease: Improving Global Outcomes

KDIGO risk stratification

The KDIGO-based heatmap analysis revealed that 28,907 patients were classified as having no CKD or the lowest risk of CKD progression. A total of 2,309 patients were identified as having a moderate risk of CKD progression, 760 patients (orange) were identified as having a high risk, and 537 patients (red) were categorised as having the highest risk of CKD progression (Figure 3).

**Figure 3:**
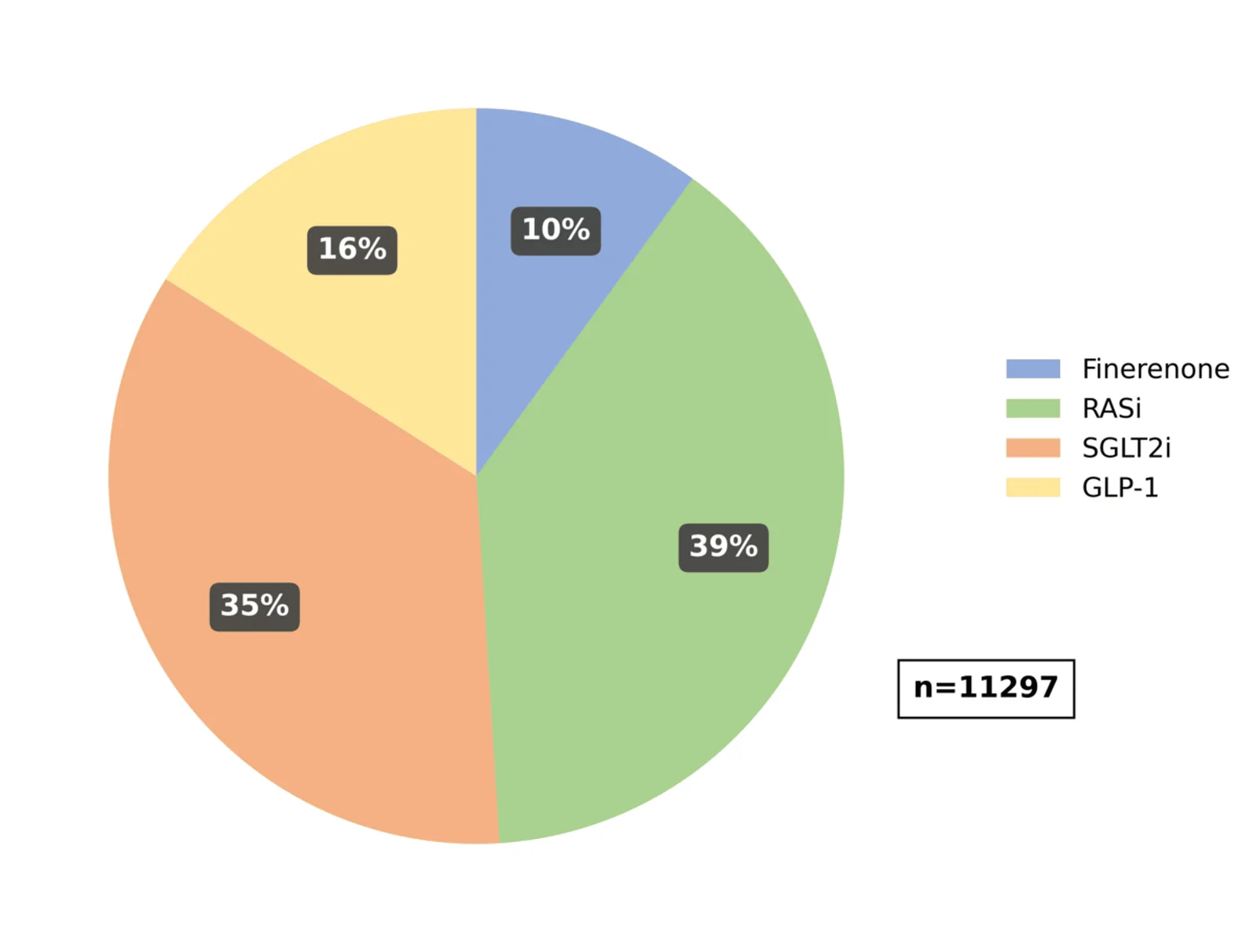
Treatment usage in high and very high-risk patients GLP-1 RAs = glucagon-like peptide-1 receptor agonists; RASi = renin-angiotensin system inhibitors; SGLT2i = sodium-glucose cotransporter 2 inhibitors

Following the classification into five CKD stages according to KDIGO guidelines, the distribution of risk levels for RASi-treated patients within each stage was assessed. Further, among the high- and very high-risk KDIGO categories, 39% (n=4406) of the patients were undergoing RASi treatment (Figure 4).

**Figure 4:**
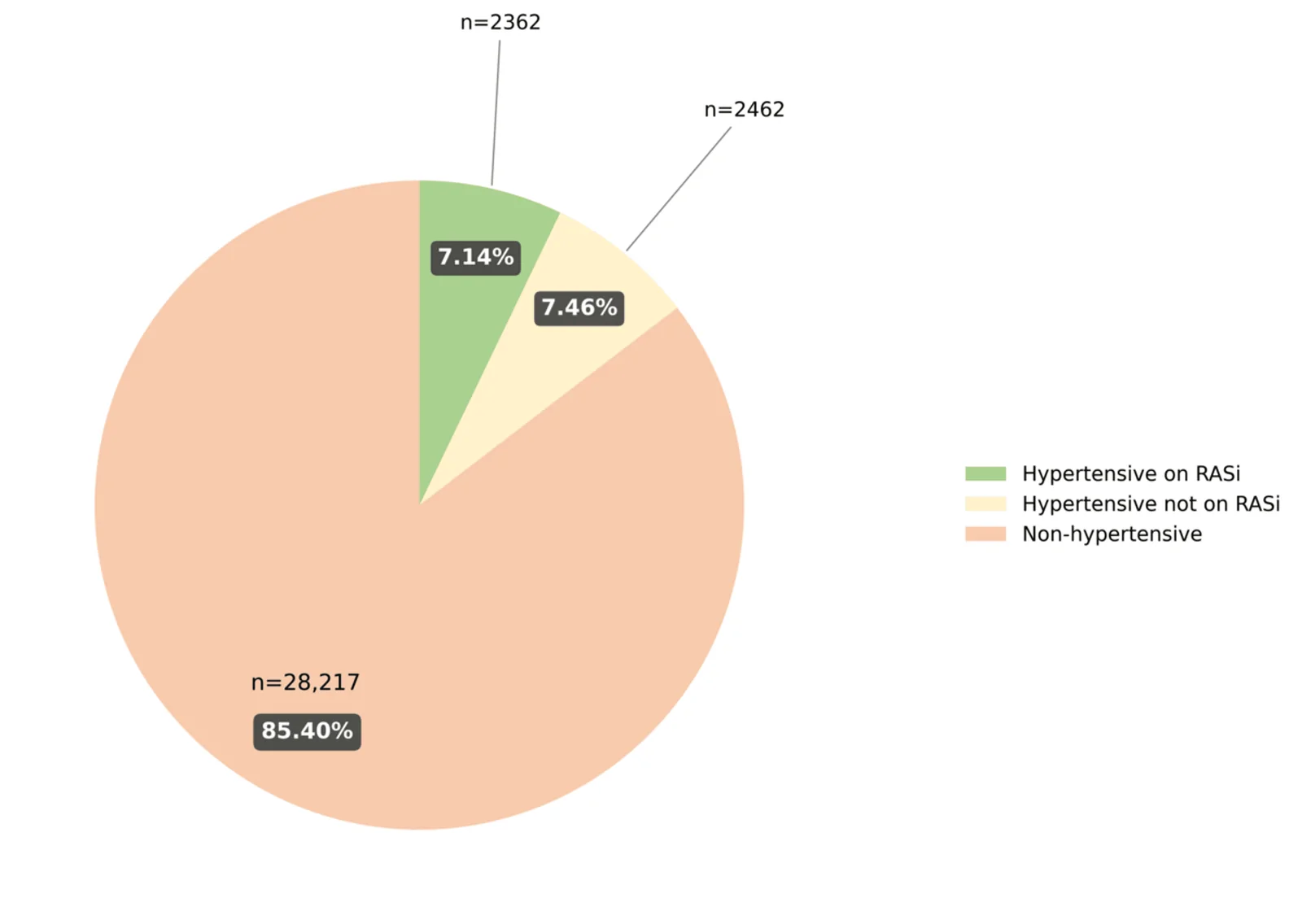
RASi utilisation among hypertensive patients RASi = renin-angiotensin system inhibitors

## Discussion

T2D remains highly prevalent on a global scale and is associated with multiple complications, including nephropathy, coronary artery disease, cerebrovascular disease, and peripheral artery disease, among others [41]. Further, an elevated albuminuria level plays a pivotal role in identifying the risk of CKD progression to kidney failure, CV risks, and premature mortality and is also considered a risk factor for CVD [42]. The KDIGO 2024 Clinical Practice Guideline for the Evaluation and Management of Chronic Kidney Disease recommends the initiation of RASi (ACEi or ARB) for diabetic individuals with DKD (G1-G4) and an elevated uACR [43]. Beyond renoprotection, RASi have also been established as effective antihypertensive agents [44]. One in four patients in the study cohort were found to have an elevated uACR, with some patients at high or very high renal risk and microalbuminuria, and some patients were also reported to be hypertensive. Recent data confirm that intensive blood pressure control in patients with T2D and hypertension reduces mortality. Despite its benefit as an effective antihypertensive treatment, RASi was utilised in less than 50% of the hypertensive population in this study cohort, highlighting a concerning gap in optimal treatment implementation [45].

The American Heart Association recently updated its guidelines on the definition and management of hypertension [46]. According to these recommendations, normal blood pressure is defined as a systolic blood pressure (SBP) of <120 mmHg and diastolic blood pressure (DBP) of <80 mmHg. Elevated blood pressure corresponds to an SBP of 120-129 mmHg with DBP <80 mmHg. Stage 1 hypertension is defined as an SBP of 130-139 mmHg or DBP of 80-89 mmHg, while Stage 2 hypertension is an SBP of ≥140 mmHg or DBP ≥90 mmHg [46]. Further, the evidence highlights the importance of optimal BP control in patients with T2DM. A recent study conducted in a Chinese population of patients with T2D and preserved kidney function demonstrated that reducing systolic BP <120 mmHg conferred additional cardiovascular benefit in terms of mortality. This trial therefore highlighted a persistent treatment gap, as only 60% of patients were receiving RASi despite their established antihypertensive and renoprotective benefits [45]. These findings are consistent with broader epidemiological data such as the Cardiovascular Consortium demonstrating BP as the single most important determinant of mortality in patients >50 years of age, across both sexes, reinforcing the need for comprehensive implementation of guideline-directed antihypertensive therapies [47].

According to a US-based study of 15,400 diabetic patients with CKD, patients were not on an ACE inhibitor or ARB at the time of CKD identification. Of these, only 17% began RASi therapy within the subsequent year [48]. Similarly, in Medicare Part D beneficiaries, only 43% of patients with both diabetes and CKD received an ACEi or ARB, with the rate rising to around 63% when hypertension was also present [49]. In a Canadian registry, 49% of diabetic patients had CKD, and only 33% were treated with an ACEi or ARB [50].

Adverse effects such as hyperkalemia, acute kidney failure, and significant BP reductions are associated with RASi, becoming a barrier to patients’ treatment [36]. Several studies suggest that maintaining or appropriately adjusting RASi therapy can be beneficial. For instance, discontinuing RASi due to hyperkalemia has been linked to increased risks of CV events and death, whereas continuing or increasing the dose may reduce the likelihood of cardiorenal events [19,51]. This underscores the importance of carefully managing RASi therapy to maximise its benefits while mitigating risks [19].

The introduction of novel potassium binders, called "RAS enablers", such as patiromer and sodium zirconium cyclosilicate (SZC), has reshaped hyperkalemia management. By controlling potassium levels, these agents enable patients to continue RASi therapy while maintaining its cardio-renal benefits. This advancement supports a shift in clinical practice toward proactive hyperkalemia management instead of early discontinuation of RASi [52]. These concerns regarding hyperkalemia and acute kidney injury, especially in advanced CKD, cause many clinicians to avoid or discontinue RASi. The requirement for follow-up labs to monitor potassium and creatinine is often seen as burdensome in busy clinical settings [53]. Bhandari et al. found that RASi therapy can be safely continued in patients with GFR <30 mL/min, offering benefits without accelerating renal decline [51]. Further, a meta-analysis has demonstrated the protective effect of RASi in patients with advanced kidney disease [54].

Further, suboptimal RASi usage may also be due to gaps in guideline implementation, lack of decision-support tools, high costs, limited multidisciplinary care, and limited specialist input. Additionally, various contraindications, intolerance, prior adverse reactions, pregnancy [55], and restricted access to routine laboratory monitoring and albuminuria screening may result in lower prescribing rates [56].

As a single-centre, cohort study, the findings in this study may have limited generalizability; therefore, further multi-centric study with diverse ethnic groups are warranted to validate these results. Further, the appropriateness of medication could not be fully assessed, as contraindications, intolerance information and factors affecting clinician decision-making were not available within this retrospective dataset.

## Conclusions

For patients with T2D and coexisting hypertension or albuminuria, RASi remain the cornerstone of therapy, with strong evidence supporting their benefit. This extensive real-world cohort analysis revealed that RASi prescription in patients with hypertension and/or albuminuria was inconsistent with guideline recommendations. These findings further highlight a substantial treatment gap in aligning real-world practice with international standards accepted globally.

Bridging this gap will involve a multipronged approach which includes improving awareness among clinicians, enhancing patient adherence, expanding access to monitoring and supportive therapies, and incorporating novel potassium binders to mitigate hyperkalemia-related discontinuation. In parallel, the integration of complementary therapies (such as SGLT2 inhibitors, finerenone, and GLP-1 receptor agonists) can provide additive benefits when used alongside RASi, demonstrating potential to significantly improve renal and cardiovascular outcomes in high-risk patients with T2D.
